# Comparative analyses of host responses upon infection with moderately virulent Classical swine fever virus in domestic pigs and wild boar

**DOI:** 10.1186/1743-422X-11-134

**Published:** 2014-07-29

**Authors:** Anja Petrov, Ulrike Blohm, Martin Beer, Jana Pietschmann, Sandra Blome

**Affiliations:** 1 Institute of Diagnostic Virology, Friedrich-Loeffler-Institut, Suedufer 10, 17493 Greifswald, Insel Riems, Germany; 2 Institute of Immunology, Friedrich-Loeffler-Institut, Suedufer 10, 17493 Greifswald, Insel Riems, Germany

**Keywords:** Classical swine fever virus, Host responses, Pathogenesis, Host factors

## Abstract

**Background:**

Classical swine fever (CSF) is one of the most important viral diseases of pigs. Clinical signs may vary from almost inapparent infection to a hemorrhagic fever like illness. Among the host factors leading to different disease courses are age, breed, and immune status. The aim of this study was to compare host responses of different pig breeds upon infection with a recent moderately virulent CSF virus (CSFV) strain, and to assess their impact on the clinical outcome and the efficiency of immune responses. To this means, two domestic pig types (German Landrace and hybrids), were compared to European wild boar. Along with clinical and pathological assessments and routine virological and serological methods, kinetics of immune-cellular parameters were evaluated.

**Findings:**

All animals were susceptible to infection and despite clinical differences, virus could be detected in all infected animals to similar amounts. All but one animal developed an acute disease course, two landrace animals recovered after a transient infection. One wild boar got chronically infected. Changes in the percentages of lymphocyte subsets in peripheral blood did not show a clear correlation with the clinical outcome. High and early titers of neutralizing antibodies were especially detected in wild boar and German Landrace pigs.

**Conclusions:**

While differences among breeds did not have the expected impact on course and outcome of CSFV infection, preload with facultative pathogens and even small differences in age seemed to be more relevant. Future studies will target the characterization of responses observed during different disease courses including cytokine reactions and further analyses of lymphocyte subsets.

## Findings

Clinical signs of classical swine fever (CSF) can range from an almost inapparent infection to a hemorrhagic fever like illness with high mortality. Factors influencing disease severity and outcome include the virulence of the CSF virus (CSFV) isolate as well as the age and immune status of the host [1-3]. However, neither beneficial nor detrimental host reaction patterns have been defined up to know, and the influence of breed-related factors remains unclear. Yet, indications exist that breed and race may have a relevant impact on the severity of the disease [1,4-6]. To target this issue, the presented study was undertaken to compare host responses of different pig breeds upon infection with a recent moderately virulent CSFV strain.

Six German landrace pigs (12 weeks of age), six hybrid pigs (8–10 weeks of age), and six European wild boar (12 weeks of age), were oronasally inoculated with 10^5.5^ tissue culture infectious doses 50% of the moderately virulent CSFV strain “Roesrath” (CSF1045). Three additional pigs of each breed acted as negative controls (housed separately). Clinical scores (CS) were assessed as previously described [7], and rectal body temperatures were recorded. While body temperatures of domestic pigs could be assessed daily, wild boar were measured upon blood collection only as they did not tolerate measurement without restraint. All animals were subjected to necropsy. For the execution of the experiment, all applicable animal welfare regulations, including EU Directive 2010/63/EC and institutional guidelines, were taken into consideration. The animal experiment was approved by the competent German authority (Landesamt für Landwirtschaft, Lebensmittelsicherheit und Fischerei Mecklenburg-Vorpommern) under reference number 7221.3-1.1-015/12.

Blood samples were collected in regular intervals from 0 to 28 days post inoculation (dpi). Peripheral blood mononuclear cells (PBMC) were subjected to multicolor immuno-staining for flow cytometry analysis of pig-cell surface markers using a BD FACSCanto™ flow cytometer (BD Biosciences). Virus isolation and neutralization tests (NT) were carried out as previously described [8]. All methodological details can be obtained from the author’s upon request.

**Figure 1:**
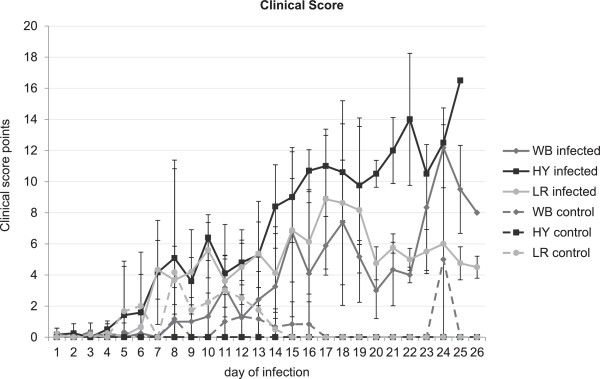
**Group mean values (mean value ± standard deviation) for clinical score points of European wild boar (WB), commercial fattening hybrids (HY) and German Landrace (LR) pigs.** Each race/breed was divided into one group for infection with CSFV “Roesrath” (infected) and one group acting as negative control (control). During the cause of disease total numbers of pigs decreased due to euthanasia.

Landrace pigs developed first clinical signs at 3 dpi. While four animals developed an acute-lethal course of the disease with severe clinical symptoms (see Figure 1 and Additional file 1: Table S1), two animals recovered. Clinical scores mirrored the disease outcome (see Figure 1) and mortality reached 66%. Post mortem examinations revealed CSF symptoms in all pigs with acute-lethal infection (see Additional file 1: Table S1). The surviving animals (LR#56 and LR#59) showed poor nutritional status and multifocal petechiae in the kidney.

Hybrid pigs showed first clinical signs from 3 dpi that worsened till the day of euthanasia (see Figure 1 and Additional file 1: Table S1). All animals succumbed to infection. In post-mortem examinations, all hybrid pigs showed typical CSF lesions and severe secondary infections.

In infected wild boar, first clinical signs were observed from 5 dpi, but raises in body temperature were only sporadically observed (see Additional file 2: Table S2). In total, 5 animals showed an acute-lethal disease course, while one animal survived till the end of the trial. Thus, mortality amounted to 83%. Post-mortem examinations revealed severe pathological lesions, both CSF specific and related to secondary infections (see Additional file 1: Table S1).

**Figure 2:**
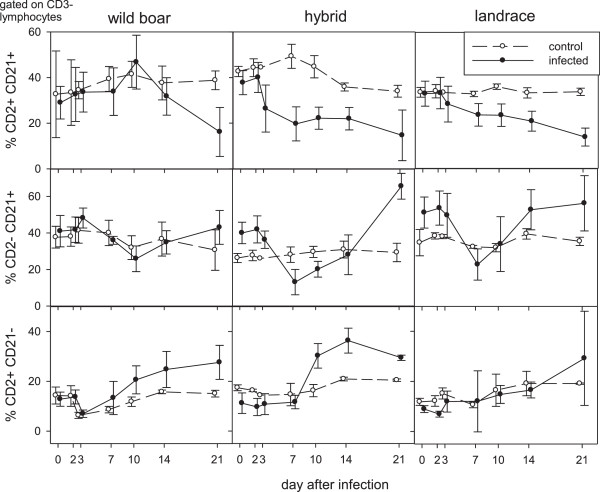
**B-cell related responses upon infection.** Blood lymphocytes were immune-stained to determine the frequency of different B cell subpopulations by FACS analysis: conventional B cells: CD3-CD2 + CD21+; activated B cells upon antigen contact: CD3-CD2-CD21+ and antibody forming and/or memory B cells: CD3-CD2 + CD21-. Filled symbols represent data from infected animals in comparison to uninfected controls (open symbols).

**Figure 3:**
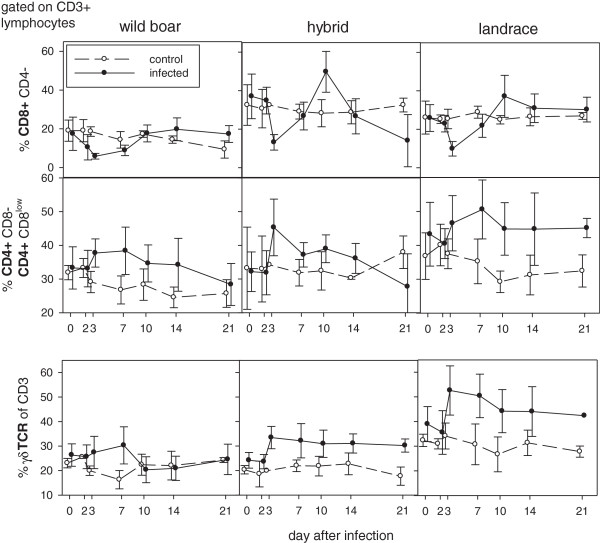
**T-cell related responses upon infection.** Percentage of T cell subpopulations of blood lymphocytes is given: cytotoxic T cells: CD8 + CD4-; T-helper cells/memory T-helper cells: CD4 + CD8-/CD4 + CD8low. Bottom row shows percentage of γδTCR + T cells of all T cells during infection. Filled symbols represent data from infected animals in comparison to uninfected controls (open symbols).

In the group of control pigs, unspecific symptoms were occasionally observed and led to euthanasia of one landrace pig at 7 dpi (dyspnea upon bleeding), and of one wild boar at 23 dpi (ruptured gall bladder, severe gastritis and enteritis).Parameters indicative for the B-cell populations in peripheral blood are summarized in Figure 2: The percentage of cells with CD2 + CD21+ phenotype (naïve B-cells) was down regulated in all infected groups. After an initial decline, an increase of CD2-CD21+ cells (phenotype of B-cells after activation) was observed in all infected groups (see Figure 2). Cells representing the phenotype of antibody producing plasma cells (CD2 + CD21-) showed a percentage increase in all infected groups with highest changes in hybrid pigs from 7 dpi. With regard to T-cell populations (see Figure 3), all inoculated animals showed slightly elevated CD4+ T helper cells starting from 3 dpi compared to the controls (see Figure 3). The reaction was most pronounced in landrace pigs. Following the increase of helper cells, an increase of cells with a CD8 + CD4- phenotype (cytotoxic T cells, CTL) was detectable. The highest percentage peak was observed in hybrid pigs. Furthermore, an increase in γδ-TCR-positive T cells was detectable in domestic pigs, especially in landrace pigs (see Figure 3).

**Figure 4:**
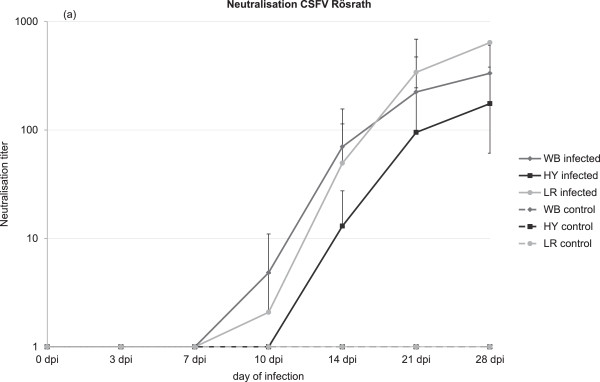
**Mean values of antibody responses of infected groups of each race (WB: wild boar; HY: commercial fattening hybrid; LR: German Landrace).** Results of the neutralization tests using CSFV strain “Roesrath” are shown in (a), in which antibody titers are represented as log_10_ ND_50_.

Virus isolation was positive for all samples from infected animals taken at 7 and 10 dpi. Thereafter, virus detection mirrored the clinical status and most tonsil samples taken at necropsy were virus isolation positive.With regard to antibody detection, landrace pigs showed one weak-positive NT result at 10 dpi (see Figure 4). At 14 dpi, neutralization tests were positive for 2 out of 4 pigs. At 21 and 28 dpi, all remaining pigs were found positive with high homologue titers in surviving pigs (see Figure 4).Hybrid pigs became positive in NTs from 14 dpi (two animals). From 21 dpi, all remaining pigs were found positive (see Figure 4).In wild boar, first antibodies were detected at 10 dpi with 2 out of 6 animals in the NTs. From 14 dpi, all tested wild boar were positive in the NTs with the homologue virus (see Figure 4).

Classical swine fever may cause most variable clinical syndromes and it is generally acknowledged that disease courses are influenced by both virus and host factors. On the host’s side, age and immune status are main parameters that influence disease course and outcome [9]. However, breed factors were also often discussed to play an important role. Depner et al. [1] showed that German landrace pigs were more severely affected than crossbred animals. Influence of breed was also seen when susceptibility was assessed in indigenous Moo Laat and improved Large White/Landrace [4]. In contrast, no differences were seen by Bunzenthal [10].

In the presented study, two domestic pig breeds were compared to European wild boar in a CSFV infection experiment. All animals proved to be susceptible to CSFV, and all but one animal enrolled in this study developed an acute course of CSF. For all hybrids and all but one wild boar, infection led to acute-lethal disease. The remaining wild boar showed both moderate antibody titers and high viral loads by the end of the trial. Based on these findings, a chronic disease course can be assumed. In the group of landrace pigs, two animals recovered after an acute-transient disease course, the others showed again an acute-lethal disease course. The clinical picture of hybrids was apparently influenced by their preload of secondary pathogens of the respiratory tract that were not sufficiently controlled by metaphylactic antibiotic treatment. Necropsy gave rise to suspicions of *Actinobacillus pleuropneumoniae* and *Haemophilus parasuis* infections. In addition to these secondary infections, hybrids were slightly younger than wild boar and landrace pigs (about two to three weeks), and the body weight was markedly lower than that of the landrace pigs. Taken together, these facts might also have influenced the clinical picture in the hybrid pigs.

In terms of serological responses, wild boar showed earliest responses. However, by the end of the trial, titers of neutralizing antibodies were similar or even higher in landrace pigs. In hybrids, E2 antibodies were only detected late in some animals and to lower titers. This reflects the clinical picture but contrasts tendencies seen in the responses of lymphocyte phenotypes (with regard to percentages of cells with plasma cell phenotype).

Despite the fact that the majority of leukocytes will be active outside the blood compartment, changes in the percentages of different lymphocyte phenotypes were investigated in blood samples as the only matrix that allowed kinetics in individual animals. With regard to B-cell responses in peripheral blood, some breed-depended patterns were observed that were however not statistically significant among the different groups. Upon infection, all animals showed a down regulation of CD2 + CD21+ cells (phenotype of naïve B-cells), this could be either due to depletion or an indication of B-cell activation. As especially domestic pigs showed an increase of cells presenting the phenotype of primed and activated B-cells (CD2-CD21+) after 7 dpi, activation could be suggested. Interestingly, the increase of cells displaying the phenotype of antibody producing plasma cells (CD2 + CD21-) was highest in hybrid pigs. This is in contrast to both clinical course and serology. However, due to the lack of additional plasma cell markers at the time point of the experiment (CD79a) and the possible impact of lymphocyte depletion, these results have to be viewed with caution and need further investigation. All investigated breeds showed slightly elevated helper cells from 3 dpi. Following the increase of helper cells, an increase of CD8 + CD4- CTLs was detectable. Strongest CTL proliferation was seen again in hybrid pigs. Preceding CTL proliferation, probably virus-mediated decrease of CD8 + CD4- T cells was detectable in all animals. This is in line with previous studies that showed that CSFV is able to suppress porcine T cells [11] and to induce killing of T cells [12]. In domestic pigs an increase of γδ TCR positive T cells was detectable, more pronounced in landrace pigs. The γδ T cells are discussed as antigen presenting cells in swine [13]. Clearly, changes in lymphocyte subsets need further investigation, especially with regard to harmful pattern and involvement of the immune system in the pathogenesis of CSF as was suggested by several authors [14-16].

While differences among breeds did not have the expected impact on course and outcome of CSFV infection, preload with facultative pathogens and even small differences in age seemed to be more relevant. Future studies will target the characterization of responses observed during different disease courses including cytokine reactions and further analyses of lymphocyte subsets.

## Competing interests

The authors declare that they have no competing interests.

## Authors’ contributions

AP carried out the animal trial, participated in the conception and design of the presented study, investigated samples from the related animal trial using serological and virological methods, performed blood count analyses, and drafted the manuscript. UB analyzed cellular responses upon infection. MB conceived the study, and participated in its design and coordination and helped to critically revise the manuscript. JP was involved in the execution of the animal trial and the related laboratory analyses. SB supervised the whole study and was involved in both the conception and execution of the animal trial. Moreover, SB critically revised the manuscript. All authors read and approved the final manuscript.

## Supplementary Material

Additional file 1: Table S1 Overview on clinical presentation and disease courses upon infection with CSFV strain “Roesrath”. Click here for file

Additional file 2: Table S2 Rectal body temperatures upon infection with CSFV strain “Roesrath” (0–28 days post infection). Fever was defined as a body temperature >40°C for at least two consecutive days. Temperatures >40°C but <40.5 are marked in yellow, temperatures >40.5°C in red. WB = wild boar, HY = hybrid pigs, LR = landrace pigs, inf = infected, ctr = negative control, nd = not determined. Click here for file
