# Supplementary material for: Comparative analyses of host responses upon infection with moderately virulent Classical swine fever virus in domestic pigs and wild boar
Source: Virol J. 2014 Jul 29;11:134. doi: 10.1186/1743-422X-11-134 (PMC4118204; doi:10.1186/1743-422X-11-134)
Supplement: Additional file 1: Table S1 — Overview on clinical presentation and disease courses upon infection with CSFV strain “Roesrath”. [file 1743-422X-11-134-S1.doc]

**Table S1: Overview on clinical presentation and disease courses upon infection with CSFV strain “Roesrath”.**

Onset refers to the occurrence of clinical signs excluding fever. Duration is given as the number of consecutive days (int. = intermittent, d = day). Unspecific symptoms (Unspec.) include depression and changes in general bearing, reduced liveliness, lack of appetite, conjunctivitis, gastro-intestinal and respiratory signs. Neurological signs (Neurol.) include ataxia, hind leg paresis, and uncontrolled shivering. Haemorrhagic signs (Haem.) refer to skin haemorrhages and cyanosis. Pathological lesions are presented for the lymphatic organs in particular (To. = tonsil, Lnn. = lymphnodes, Sp. = spleen) . Moreover, haemorrhagic lesions (Haem.) and secondary infections (Sec.Inf.) are accounted for. Additional abbreviations: WB = wild boar; HY = hybrid pigs; LR = landrace pigs; INF = inoculated animal; NC = uninfected control animal; DOE = day of euthanasia; AL = acute-lethal disease course; AT = acute-transient disease course, CH = chronic illness.

| **Animal** | **Breed** | **Status** | **Fever** | **Onset** | **Duration** | **CS max** | **Clinical Symptoms** | | | **Outcome** | **DOE** | **Pathology** | | | | | **Seroconversion** | |
| --- | --- | --- | --- | --- | --- | --- | --- | --- | --- | --- | --- | --- | --- | --- | --- | --- | --- | --- |
|  |  |  | ***[T°C* ≥*40]*** |  |  |  | ***Unspec.*** | ***Neuro.*** | ***Haem.*** |  |  | ***To.*** | ***Lnn.*** | ***Sp.*** | ***Haem.*** | ***Sec. Inf.*** | ***E2*** | ***Erns*** |
| #WB08 | WB | INF | 10 dpi | 5 dpi | 11 d | 9.0 | x |  |  | AL | 15 dpi | x | x |  |  |  | x | x |
| #WB09 | WB | INF | 12 dpi | 8 dpi | 16 d | 15.0 | x | x |  | AL | 23 dpi | x | x |  | x | x | x | x |
| #WB10 | WB | INF | int. | 5 dpi | 13 d | 13.0 | x | x |  | AL | 17 dpi | x | x |  | x | x | x | x |
| #WB13 | WB | INF | 10 dpi | 10 dpi | 15 d | 11.5 | x | x |  | AL | 24 dpi | x | x |  | x | x | x | x |
| #WB15 | WB | INF | int. | 5 dpi | 24 d | 11.5 | x | x |  | CH | 28 dpi | x | x |  |  | x | x | x |
| #WB16 | WB | INF |  | 7 dpi | 9 d | 9.5 | x |  |  | AL | 15 dpi | x | x |  |  | x | x | x |
| #WB07 | WB | NC |  |  |  |  |  |  |  |  | 28 dpi |  |  |  |  |  |  |  |
| #WB12 | WB | NC |  | 4 dpi | 1 d | 1.0 | x |  |  |  | 28 dpi |  |  |  |  |  |  |  |
| #WB14 | WB | NC |  | 2 dpi | int. | 15.0 | x | x |  |  | 23 dpi |  |  |  |  |  |  |  |
| #DP49 | HY | INF | 3-24 dpi | 3 dpi | 22 d | 16.5 | x | x | x | AL | 24 dpi | x | x | x | x | x | x | x |
| #DP50 | HY | INF | 3-18 dpi | 3 dpi | 16 d | 14.5 | x | x | x | AL | 18 dpi |  | x |  | x | x | x | x |
| #DP51 | HY | INF | 4-17 dpi | 5 dpi | 13 d | 14.0 | x | x |  | AL | 17 dpi |  | x |  |  | x | x | x |
| #DP52 | HY | INF | 3-7 dpi | 3 dpi | 5 d | 16.5 | x |  |  | AL | 7 dpi |  |  |  |  | x |  |  |
| #DP53 | HY | INF | 5-21 dpi | 5 dpi | 17 d | 17.0 | x | x | x | AL | 21 dpi |  | x |  | x | x | x | x |
| #DP54 | HY | INF | 3-19 dpi | 6 dpi | 19 d | 11.0 | x | x | x | AL | 20 dpi | x | x |  | x | x | x | x |
| #DP43 | HY | NC | 20 dpi |  |  |  |  |  |  |  | 28 dpi |  |  |  |  |  |  |  |
| #DP44 | HY | NC | int. |  |  |  |  |  |  |  | 28 dpi |  |  |  |  |  |  |  |
| #DP45 | HY | NC | int. |  |  |  |  |  |  |  | 28 dpi |  |  |  |  |  |  |  |
| #DP55 | LR | INF | 3 - 11 dpi | 6 dpi | 6 d | 8.0 | x | x |  | AL | 11 dpi |  | x |  | x |  |  | x |
| #DP56 | LR | INF | int. | 4 dpi | 25 d | 7.0 | x | x |  | AT | 28 dpi |  |  |  | x |  | x | x |
| #DP57 | LR | INF | int. | 5 dpi | 14 d | 15.0 | x | x | x | AL | 18 dpi | x | x |  | x | x | x | x |
| #DP58 | LR | INF | 3-17 dpi | 5 dpi | 13 d | 15.5 | x | x | x | AL | 17 dpi | x | x |  | x | x | x | x |
| #DP59 | LR | INF | 4-20 dpi | 6 dpi | 23 d | 6.0 | x | x |  | AT | 28 dpi |  | x |  | x | x | x | x |
| #DP60 | LR | INF | 4-7 dpi | 3 dpi | 5 d | 7.5 | x | x |  | AL | 7 dpi |  | x |  | x |  |  |  |
| #DP46 | LR | NC | 4 dpi | 4 dpi | int. | 12.5 | x | x |  |  | 7 dpi |  |  |  |  |  |  |  |
| #DP47 | LR | NC | int. | 8 dpi | 6 d | 6.0 | x | x |  |  | 28 dpi |  |  |  |  |  |  |  |
| #DP48 | LR | NC | int. |  |  |  |  |  |  |  | 28 dpi |  |  |  |  |  |  |  |
