# Supplementary material for: Comparative analyses of host responses upon infection with moderately virulent Classical swine fever virus in domestic pigs and wild boar
Source: Virol J. 2014 Jul 29;11:134. doi: 10.1186/1743-422X-11-134 (PMC4118204; doi:10.1186/1743-422X-11-134)
Supplement: Additional file 2: Table S2 — Rectal body temperatures upon infection with CSFV strain “Roesrath” (0–28 days post infection). Fever was defined as a body temperature >40°C for at least two consecutive days. Temperatures >40°C but <40.5 are marked in yellow, temperatures >40.5°C in red. WB = wild boar, HY = hybrid pigs, LR = landrace pigs, inf = infected, ctr = negative control, nd = not determined. [file 1743-422X-11-134-S2.ppt]

## Slide 1
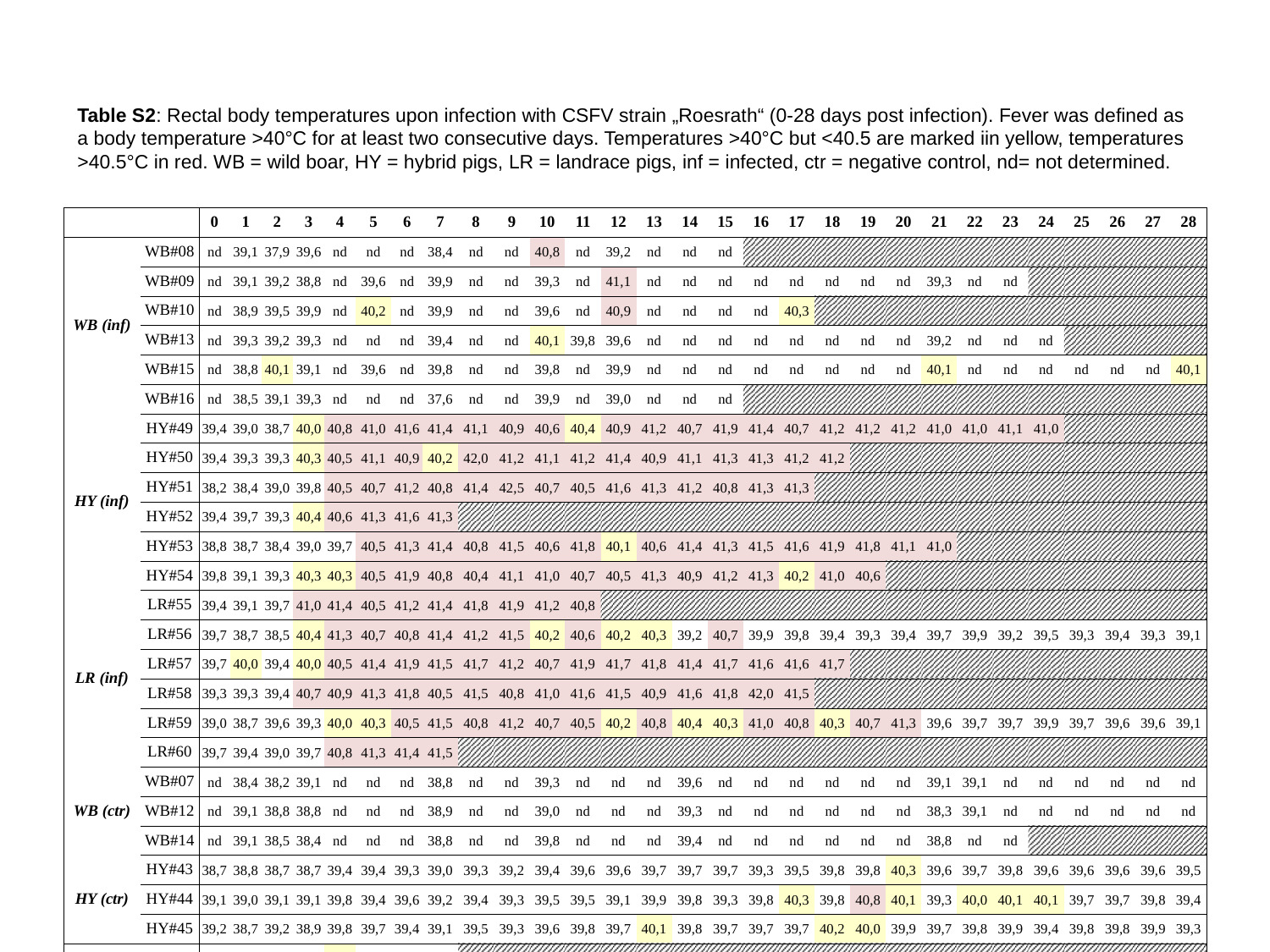

Table S2: Rectal body temperatures upon infection with CSFV strain „Roesrath“ (0-28 days post infection). Fever was defined as a body temperature >40°C for at least two consecutive days. Temperatures >40°C but <40.5 are marked iin yellow, temperatures >40.5°C in red. WB = wild boar, HY = hybrid pigs, LR = landrace pigs, inf = infected, ctr = negative control, nd= not determined.
| | | 0 | 1 | 2 | 3 | 4 | 5 | 6 | 7 | 8 | 9 | 10 | 11 | 12 | 13 | 14 | 15 | 16 | 17 | 18 | 19 | 20 | 21 | 22 | 23 | 24 | 25 | 26 | 27 | 28 |
| --- | --- | --- | --- | --- | --- | --- | --- | --- | --- | --- | --- | --- | --- | --- | --- | --- | --- | --- | --- | --- | --- | --- | --- | --- | --- | --- | --- | --- | --- | --- |
| WB (inf) | WB#08 | nd | 39,1 | 37,9 | 39,6 | nd | nd | nd | 38,4 | nd | nd | 40,8 | nd | 39,2 | nd | nd | nd | | | | | | | | | | | | | |
| | WB#09 | nd | 39,1 | 39,2 | 38,8 | nd | 39,6 | nd | 39,9 | nd | nd | 39,3 | nd | 41,1 | nd | nd | nd | nd | nd | nd | nd | nd | 39,3 | nd | nd | | | | | |
| | WB#10 | nd | 38,9 | 39,5 | 39,9 | nd | 40,2 | nd | 39,9 | nd | nd | 39,6 | nd | 40,9 | nd | nd | nd | nd | 40,3 | | | | | | | | | | | |
| | WB#13 | nd | 39,3 | 39,2 | 39,3 | nd | nd | nd | 39,4 | nd | nd | 40,1 | 39,8 | 39,6 | nd | nd | nd | nd | nd | nd | nd | nd | 39,2 | nd | nd | nd | | | | |
| | WB#15 | nd | 38,8 | 40,1 | 39,1 | nd | 39,6 | nd | 39,8 | nd | nd | 39,8 | nd | 39,9 | nd | nd | nd | nd | nd | nd | nd | nd | 40,1 | nd | nd | nd | nd | nd | nd | 40,1 |
| | WB#16 | nd | 38,5 | 39,1 | 39,3 | nd | nd | nd | 37,6 | nd | nd | 39,9 | nd | 39,0 | nd | nd | nd | | | | | | | | | | | | | |
| HY (inf) | HY#49 | 39,4 | 39,0 | 38,7 | 40,0 | 40,8 | 41,0 | 41,6 | 41,4 | 41,1 | 40,9 | 40,6 | 40,4 | 40,9 | 41,2 | 40,7 | 41,9 | 41,4 | 40,7 | 41,2 | 41,2 | 41,2 | 41,0 | 41,0 | 41,1 | 41,0 | | | | |
| | HY#50 | 39,4 | 39,3 | 39,3 | 40,3 | 40,5 | 41,1 | 40,9 | 40,2 | 42,0 | 41,2 | 41,1 | 41,2 | 41,4 | 40,9 | 41,1 | 41,3 | 41,3 | 41,2 | 41,2 | | | | | | | | | | |
| | HY#51 | 38,2 | 38,4 | 39,0 | 39,8 | 40,5 | 40,7 | 41,2 | 40,8 | 41,4 | 42,5 | 40,7 | 40,5 | 41,6 | 41,3 | 41,2 | 40,8 | 41,3 | 41,3 | | | | | | | | | | | |
| | HY#52 | 39,4 | 39,7 | 39,3 | 40,4 | 40,6 | 41,3 | 41,6 | 41,3 | | | | | | | | | | | | | | | | | | | | | |
| | HY#53 | 38,8 | 38,7 | 38,4 | 39,0 | 39,7 | 40,5 | 41,3 | 41,4 | 40,8 | 41,5 | 40,6 | 41,8 | 40,1 | 40,6 | 41,4 | 41,3 | 41,5 | 41,6 | 41,9 | 41,8 | 41,1 | 41,0 | | | | | | | |
| | HY#54 | 39,8 | 39,1 | 39,3 | 40,3 | 40,3 | 40,5 | 41,9 | 40,8 | 40,4 | 41,1 | 41,0 | 40,7 | 40,5 | 41,3 | 40,9 | 41,2 | 41,3 | 40,2 | 41,0 | 40,6 | | | | | | | | | |
| LR (inf) | LR#55 | 39,4 | 39,1 | 39,7 | 41,0 | 41,4 | 40,5 | 41,2 | 41,4 | 41,8 | 41,9 | 41,2 | 40,8 | | | | | | | | | | | | | | | | | |
| | LR#56 | 39,7 | 38,7 | 38,5 | 40,4 | 41,3 | 40,7 | 40,8 | 41,4 | 41,2 | 41,5 | 40,2 | 40,6 | 40,2 | 40,3 | 39,2 | 40,7 | 39,9 | 39,8 | 39,4 | 39,3 | 39,4 | 39,7 | 39,9 | 39,2 | 39,5 | 39,3 | 39,4 | 39,3 | 39,1 |
| | LR#57 | 39,7 | 40,0 | 39,4 | 40,0 | 40,5 | 41,4 | 41,9 | 41,5 | 41,7 | 41,2 | 40,7 | 41,9 | 41,7 | 41,8 | 41,4 | 41,7 | 41,6 | 41,6 | 41,7 | | | | | | | | | | |
| | LR#58 | 39,3 | 39,3 | 39,4 | 40,7 | 40,9 | 41,3 | 41,8 | 40,5 | 41,5 | 40,8 | 41,0 | 41,6 | 41,5 | 40,9 | 41,6 | 41,8 | 42,0 | 41,5 | | | | | | | | | | | |
| | LR#59 | 39,0 | 38,7 | 39,6 | 39,3 | 40,0 | 40,3 | 40,5 | 41,5 | 40,8 | 41,2 | 40,7 | 40,5 | 40,2 | 40,8 | 40,4 | 40,3 | 41,0 | 40,8 | 40,3 | 40,7 | 41,3 | 39,6 | 39,7 | 39,7 | 39,9 | 39,7 | 39,6 | 39,6 | 39,1 |
| | LR#60 | 39,7 | 39,4 | 39,0 | 39,7 | 40,8 | 41,3 | 41,4 | 41,5 | | | | | | | | | | | | | | | | | | | | | |
| WB (ctr) | WB#07 | nd | 38,4 | 38,2 | 39,1 | nd | nd | nd | 38,8 | nd | nd | 39,3 | nd | nd | nd | 39,6 | nd | nd | nd | nd | nd | nd | 39,1 | 39,1 | nd | nd | nd | nd | nd | nd |
| | WB#12 | nd | 39,1 | 38,8 | 38,8 | nd | nd | nd | 38,9 | nd | nd | 39,0 | nd | nd | nd | 39,3 | nd | nd | nd | nd | nd | nd | 38,3 | 39,1 | nd | nd | nd | nd | nd | nd |
| | WB#14 | nd | 39,1 | 38,5 | 38,4 | nd | nd | nd | 38,8 | nd | nd | 39,8 | nd | nd | nd | 39,4 | nd | nd | nd | nd | nd | nd | 38,8 | nd | nd | | | | | |
| HY (ctr) | HY#43 | 38,7 | 38,8 | 38,7 | 38,7 | 39,4 | 39,4 | 39,3 | 39,0 | 39,3 | 39,2 | 39,4 | 39,6 | 39,6 | 39,7 | 39,7 | 39,7 | 39,3 | 39,5 | 39,8 | 39,8 | 40,3 | 39,6 | 39,7 | 39,8 | 39,6 | 39,6 | 39,6 | 39,6 | 39,5 |
| | HY#44 | 39,1 | 39,0 | 39,1 | 39,1 | 39,8 | 39,4 | 39,6 | 39,2 | 39,4 | 39,3 | 39,5 | 39,5 | 39,1 | 39,9 | 39,8 | 39,3 | 39,8 | 40,3 | 39,8 | 40,8 | 40,1 | 39,3 | 40,0 | 40,1 | 40,1 | 39,7 | 39,7 | 39,8 | 39,4 |
| | HY#45 | 39,2 | 38,7 | 39,2 | 38,9 | 39,8 | 39,7 | 39,4 | 39,1 | 39,5 | 39,3 | 39,6 | 39,8 | 39,7 | 40,1 | 39,8 | 39,7 | 39,7 | 39,7 | 40,2 | 40,0 | 39,9 | 39,7 | 39,8 | 39,9 | 39,4 | 39,8 | 39,8 | 39,9 | 39,3 |
| LR (ctr) | LR#46 | 39,3 | 39,1 | 39,0 | 38,5 | 40,0 | 39,6 | 39,0 | 38,3 | | | | | | | | | | | | | | | | | | | | | |
| | LR#47 | 39,0 | 39,1 | 38,8 | 39,2 | 39,5 | 39,8 | 39,7 | 39,5 | 40,1 | 40,1 | 40,1 | 40,2 | 39,5 | 39,4 | 39,5 | 39,8 | 40,8 | 40,6 | 39,9 | 40,1 | 40,5 | 40,5 | 39,9 | 40,1 | 40,1 | 39,2 | 38,8 | 39,2 | 38,8 |
| | LR#48 | 39,3 | 38,4 | 39,3 | 39,5 | 39,8 | 40,6 | 39,3 | 39,6 | 39,1 | 39,1 | 39,3 | 39,1 | 39,2 | 39,4 | 39,5 | 39,0 | 39,3 | 38,9 | 39,3 | 39,5 | 39,7 | 39,6 | 39,4 | 40,3 | 40,1 | 39,2 | 39,8 | 39,6 | 39,5 |
